# Complete Genomic Sequence of a Novel Porcine Circovirus 2 Strain, CC12

**DOI:** 10.1128/genomeA.00318-14

**Published:** 2014-04-17

**Authors:** Zeli Xing, Lisai Zhu, Chuanchuan Jia, Changming Guo, Xiaochun Gai, Lianzhi Mu, Xinping Wang

**Affiliations:** College of Veterinary Medicine, Jilin University, Changchun, Jilin, China

## Abstract

The genome sequence of a novel porcine circovirus 2 strain (CC12) is composed of 1,767 nucleotides, with two major open reading frames (ORFs). ORF1 encodes two replication-associated proteins (Rep and Rep′) with the unique mutation N186S, and ORF2 encodes a viral capsid protein (Cap) with two rare mutations, R59K and A190T.

## GENOME ANNOUNCEMENT

Porcine circovirus 2 (PCV2), a single-stranded circular DNA virus and a member of the virus family *Circoviridae*, is etiologically associated with postweaning and multisystemic wasting syndrome (PMWS), porcine dermatitis and nephropathy syndrome, porcine respiratory disease complex, granulomatous enteritis, necrotizing lymphadenitis, reproductive failure, exudative epidermitis, and congenital tremors (1–8). PCV2 infection was first reported in Canada in 1997 and later identified in the United States, France, Japan, and other countries (9, 10). Recently, this infection has become one of the most severe diseases affecting pig production in China (11–14).

PCV2 has been isolated from many countries, and three major genotypes (PCV2a, 2b, and 2c) have been proposed based on the genetic variations of isolates (13–20). PCV2b was demonstrated to be the prevalent form with an enhanced pathogenicity. We have isolated a PCV2 strain, named CC12, from a pig farm with an outbreak of PMWS in the Jilin province of China. Sequencing the genome of CC12 strain showed that it contains 1,767 nucleotides and consists of two major open reading frames (ORFs), ORF1 and ORF2. ORF1 encodes two replication-associated proteins (Rep and Rep′), and ORF2 encodes a viral capsid protein (Cap). Phylogenetic analysis showed that the CC12 strain is clustered to PCV2b, a predominant genotype affecting the world’s swine industry since 2003. Alignment analysis by the Clustal W method revealed a very unique amino acid mutation/substitution from asparagine to serine (N186S) in the Rep protein and two rare mutations in the Cap protein, one a change from arginine to lysine (R59K) and the other from alanine to threonine (A190T), in relation to PCV2 genomic sequences in GenBank. Whether these novel mutations are of significance in altering either the biological function of ORF1-encoded Rep protein or the antigenicity of the ORF2-encoded Cap protein is not clear. This will be the subject of a future investigation.

Our findings revealed a novel PCV2 strain with a unique mutation in its Rep protein and two rare mutations in the Cap protein. These data will facilitate the future investigation of the molecular pathogenesis of PCV2 and contribute to the elucidation of the functions of PCV2 proteins.

### Nucleotide sequence accession number.

The complete genome sequence of CC12 has been deposited at DDBJ/EMBL/GenBank under the accession no. KC859451.
